# Mechanism of bactericidal efficacy against nosocomial pathogenic *Staphylococcus aureus* strain caused by fatty acids from *Hermetia illucens* larvae fat

**DOI:** 10.1038/s41598-025-15858-0

**Published:** 2025-08-19

**Authors:** Heakal Mohamed, Elena Marusich, Margarita Pustovalova, Sergey Leonov

**Affiliations:** 1 Agricultural Research Center (ARC), Plant Protection Research Institute (PPRI), Dokki, Giza, Egypt; 2 Institute of Future Biophysics, Moscow Center for Advanced Studies, Moscow, 123592 Moscow, Russian Federation; 3 Institute of Cell Biophysics, Russian Academy of Sciences, Moscow Region, 142290 Pushchino, Russian Federation

**Keywords:** *Hermetia illucens*, AWME3 extract Fatty acids, MDR bacteria, Salt tolerance, SEM, TEM, Drug discovery, Microbiology

## Abstract

*Hermetia illucens (HI)* is a promising insect that widely employed as a sustainable source of food and has been recently used as a successful antimicrobial agent. Fatty acids extracted sequentially from HI larvae fight against MDR nosocomial pathogenic bacteria such as *Staphylococcus aureus*. This strain is resistant to various antibiotics, causing many issues and deaths in healthcare sectors. The present study aimed to elucidate the mechanism of bactericidal efficacy of fatty acids (FAs) in HI larvae fat against *S. aureus* ATCC 55804 strain. The disk diffusion assay, minimum inhibitory concentration (MIC), minimum bactericidal concentration (MBC), and half of the minimum inhibitory concentration (MIC50) applied in this study, proved the antimicrobial activity of fatty acids. The mechanism of FAs action was evaluated by several approaches, including inhibition of the bacterial growth curves and salt tolerance assays, scanning electron (SEM) and transmission electron (TEM) microscopies. *S. aureus* ATCC 55804 was resistant to 30% out of ten tested antibiotics belonging to different classes. In addition, microscopic observations showed the inhibitory effect of acidic water methanol extract (AWME3) by targeting of the *S. aureus* ATCC 55804 cell membrane and causing the considerable morphological alterations on the bacterial wall and destruction its cytoplasmic contents leading to the cellular content release and cell death. This study revealed the potential efficacy of AWME3 as a novel therapeutic antibacterial agent effective against resistant nosocomial bacterial pathogens.

## Introduction

Antibiotic resistance is the leading global health concern to date. The abuse of antibiotics in the medical, veterinary, and agricultural aspects, including the inadequate prescribing of antibiotics, their overuse in the livestock sector, and deficient hygiene practices in hospitals, all enhance antimicrobial resistance (AMR). AMR points to bacteria and other microorganisms’ capacity to withstand the effect of an antibiotic to which they were previously susceptible, allowing germs to survive and thrive^1^. Resistant bacteria strains display several mechanisms to combat antibiotics, including natural and acquired resistances. AMR mechanisms may be classified into four categories, including drug uptake limitation, drug target modulation, drug inhibition, and drug efflux^2^. Based on the structural differences and others, Gram-negative bacteria can use all four mechanisms, while Gram-positive bacterial cell walls do not have lipopolysaccharides in the outer membrane, thus they rarely use limited drug uptake or drug efflux mechanisms^3^. Biofilm formation is another mechanism that helps in the colonization of bacteria; further, the matrix of biofilm involves polysaccharides, proteins, and DNA, making antimicrobial agents difficult to enter the bacteria and thereby providing extensive defences^4,5^.

Black soldier fly (*Hermetia ilucens*) larvae commonly contain a high amount of fat (up to 40%) and are rich in saturated fatty acids (SFA), such as stearic (C18:0), palmitic (C16:0), myristic (C14:0), and lauric acid (C12:0). Furthermore, lauric acid, which is associated with a melanin-chitosan complex, has a wide spectrum of antimicrobial activity against *Aspergillus niger*, *Candida albicans, Salmonella* spp., and *S. aureus*^6–9^. *Hermetia ilucens* (HI) larva contains low amounts of monounsaturated (MUFA) and polyunsaturated (PUFA) fatty acids, and the spectrum of acids is very similar to that of palm kernel fat and coconut oil^10^ The antibacterial efficacy of *Hermetia illucens* lipids was evaluated against *Micrococcus flavus* and *Escherichia coli*^11^. The fatty acids profile of HI larvae depends on the chemical compounds of their rearing substrate^12,13^. HI larvae could be used for the sustainable production of proteins and lipids. Furthermore, the larvae fat has bioactive substances, especially fatty acids, which could be promising candidates to fight against microbial pathogens distributed in agriculture, veterinary, and medical fields^14–16^.

Fatty acids are the most common essential biomolecules in different biological systems and have many important biological functions, including the regulation of cell membrane functions and the activity of enzymes inside the cell. They are carboxylic acids that contain saturated or unsaturated aliphatic hydrocarbon chains. FAs can be classified into four groups as follows: (1) short-chain fatty acids (< C6), (2) medium-chain fatty acids (C6–C12), (3) long-chain fatty acids (C13–C21), and (4) very long-chain fatty acids (≥ C22), based on the chain length. In addition, FAs may have various numbers of double bonds located in different positions in their aliphatic chain, which can form large numbers of isomeric fatty acid families, e.g., geometric isomers and structural isomers. FAs exist as free fatty acid (FFA) forms in biological systems; however, they can be presented in bound forms, such as cholesterol and phospholipids, so the total fatty acids include FFAs and bound fatty acids^16^.

Antimicrobial lipids, especially fatty acids and monoglycerides, are promising antibacterial compounds that impair bacterial cell membranes, causing a wide range of inhibitory effects^17^. These two classes of antimicrobial lipids received more attention due to the pioneering studies, which focused on the antibacterial efficacy of fatty acids and different chain lengths of monoglycerides^18^. The antimicrobial potency of fatty acids based on the chain length (odd or even number of carbon atoms), the degree of unsaturation, and the number and orientation (*cis*- or *trans*-) of double bonds in the carbon backbone. The mechanisms of the antibacterial activity of fatty acids and their derivatives, specifically monoglycerides, have been examined by targeting bacterial cell membranes and leading to the disruption of their pivotal role in the cellular protection and function. Amphipathic properties of fatty acids and monoglycerides cause the destruction of biophysical phenomena of bacterial cells such as membrane impairment and pore formation. In particular, its membrane-destabilizing activity increases cell permeability and cell lysis, leading to the inhibition of bacterial culture growth (bacteriostatic action) or killing of the bacterial cells (bactericidal action)^17,19^.

Among the essential processes involved in bacterial cell membranes, two important processes include the chain of electron transport and oxidative phosphorylation, which are important for energy production in bacterial cells. These two processes are interconnected, and fatty acids have the potential to retard the electron transport chain process by connecting with electron carriers or modifying the membrane integrity, as well as interfering with oxidative phosphorylation by reducing the membrane potential and proton gradient. Moreover, fatty acids can directly inactivate membrane enzymes, especially glucosyltransferase, probably because of the similarity between fatty acids and small molecules. The interaction of antimicrobial fatty acids with bacterial cell membranes can disrupt the membrane and increase membrane permeability, thereby inducing leakage of cytosolic components. In utmost cases, increased permeability and membrane destabilization are most likely leading to cell lysis^18^. Electron microscopy techniques are promising tools to observe directly the morphological alterations that happen in bacterial cell membranes, and they are utilized to image bacterial specimens after exposure to antimicrobial lipids.

This study aims to clarify the expected mechanisms of the mixture of SFA and USFA in AWME3 extracted from HI larvae fat using several approaches (MIC, MIC50, MBC, growth curves, bacterial cell viability, salt tolerance, alteration in the bacterial cell morphology, and compartments via microscopy techniques).

## Materials and methods

### Biomaterials and reagents

For preparation of AWME3, fat was isolated from 15 days old *Hermetia illucens* (HI) larvae using a mechanical pressing machine, which was provided by NordTechSad, LLC (Arkhangelsk, Russian Federation). *Staphylococcus aureus* ATCC 55804 strain was purchased from the American Type Culture Collection (ATCC), Manassas, USA.

Antimicrobial susceptibility disks (6 mm) loaded with 100 U/mL-100 µg/mL/disk penicillin–streptomycin (P/S), 100 µg/mL/disk, 1000 µg/mL/disk chloramphenicol (Ch), 200 µg/mL/disk gentamycin (G) and 25 µg/mL/disk amphotericin B (Am)) were purchased from Gibco, Thermo Fisher Scientific, Waltham, MA, USA. Discs loaded with 2 U/disk penicillin (P), 5 µg/disk vancomycin (VA), 60 µg/disk erythromycin (E), 15 µg/disk rifampicin (RD), 1000 µg/disk kanamycin (K), and 10 µg/disk colistin (CT) were purchased from Oxoid, Basingstoke, Hampshire, United Kingdom. Different chemical reagents used in this study, including hydrochloric acid, acetic acid, methanol, ethanol, glutaraldehyde, phosphate buffer saline, osmium tetroxide, epoxy resin, and uranyl acetate, which were purchased from Thermo Fisher Scientific, Waltham, MA, USA.

### Fatty acids extraction

The third sequential acidic water methanol extract (AWME3) was extracted from the fat of HI larvae from the 3 g of HI larvae fat, following the protocol previously described.

### Fatty acids composition of AWME3 extract

The FFAs constituents of AWME3 extract were identified by GC–MS method adapted from our previous publication^15^. Briefly, the GC–MS-QP2010 ultra mass spectrometer (Shimadzu, Canby, CA, USA) equipped with an autosampler and interfaced to a mass spectrometer was used for GC–MS data acquisition. The sample separation was performed on a non-polar DB-5 ms capillary column (30 m × 0.25 mm i.d., 0.25 µm film thickness; Restek, USA), with helium as the carrier gas at a linear flow rate of 1.0–15.0 mL/min under a column head pressure of 50.4 kPa. The AWME3 1 µL of sample was injected by the autosampler injector automatically. The injector and detector temperatures maintained at 280 °C and 250 °C, respectively. The temperature program was initially set at 40 °C, held for 1 min, and then it was increased to 210 °C at a rate 15 °C/min, held for 0 min, then it was increased to 216 °C with a rate 5 °C/min and held for 0 min. Then, the temperature was increased to 300 °C with a flow rate of 40 °C/min and held for 14.87 min. The GC–MS values were analysed using electron impact ionization at 70 Ev. The content of AWME3 was identified based on a comparison of their retention time (min), peak area, peak height, and mass-spectral patterns with those spectral databases of authentic compounds stored in the National Institute of Standards and Technology (NIST) library. Compounds with chromatogram peaks matched with similarity index (SI) ≥ 70% in NIST-8 library were ascertained.

## Determination of the antimicrobial properties of AWME3 against *S. aureus* ATCC 55804 strain

### Disk diffusion assay

The susceptibility of AWME3 was determined by measuring the inhibition zone diameter (IZD) formatted by AWME3 extract treatment of bacteria in the range of 1.2–20 mg/mL concentrations. Briefly, Mueller–Hinton agar (HM) plates were seeded with *Staphylococcus aureus* ATCC 55804 strain, which was incubated overnight at 37 °C and shaking at 200 rpm/min, and then adjusted to 0.5 McFarland standard (1 × 10^8^ CFU/mL) of bacterial density^20^. Then, 50 µL of 1.25, 2.5, 5, 10, and 20 mg/mL of AWME3 extract concentrations were introduced to standard sterile 6 mm paper disks. The 50 µL of acetic-water–methanol (AWM) extraction reagent was used as a negative control. 50 µL of P/S (200 U/mL-200 μg/mL) was used as a positive control. Finally, all disks were dried under ambient and sterile conditions, then transferred and fixed gently on the surface of the streaked plates and incubated at 37 °C for 24 h. All samples were applied in duplicates in three independent experiments.

## Determination of minimum inhibitory concentration (MIC) and the minimum bactericidal concentration (MBC)

The minimum inhibitory concentration (MIC) was determined as the lowest concentration of AWME3 and P/S that shows a visible bacterial growth inhibition after overnight incubation. The minimum bactericidal concentration (MBC) was defined as complete inhibition of bacterial growth by tested antimicrobials after overnight incubation. Both assays were performed by broth microdilution methods as previously described^20^. Briefly, in a 96-well plate, 100 µL of the AWME3 extract was diluted in LB broth by twofold dilutions to give a final concentration at a range (48–3000 mg/mL) after adding 100 µL of the bacterial suspension (~ 10^6^ CFU/ml). Likewise, the positive control (P/S) was serially diluted by a twofold dilution method to give the final concentration at range (0.6–78.1 µg/mL). Microplates were sealed and incubated at 37 °C for 24 h with shaking at 200 rpm/min. Wells with no visible bacterial growth were considered as MIC values. To determine the MBC value, 10 μL culture from the well with MIC value and were undergo to the serial of dilutions, then spotted on MH agar plates, and incubated for another 48 h at 37 °C. The complete absence of growth was considered as the MBC value. All MIC and MBC assays were performed in triplicates in three different sets of experiments.

### Alamar blue assay

The influence of AWME3 on bacterial cell viability was carried out in a 96-well plate using Alamar blue (AB) cell viability assay kit efficacy (Thermo Scientific, Loughborough, UK)^21,22^. For the assay, 100 µL of AWME3 were twofold serially in MH broth medium to get the final 1.5, 0.75, 0.38, 0.19, 0.095, 0.048, 0.024, and 0.012 mg/mL concentrations of AWME3. Then, 100 µL of bacteria with a density of 10^6^ CFU/mL of *S. aureus* ATCC 55804 was added to the plate, the plate was sealed to avoid evaporation, and incubated for 24 h at 37 °C. Similarly, the positive control (P/S) was evaluated at the range of 0.6–78.1 μg/mL concentrations against *S. aureu*s ATCC 55804 followed by incubation at the same setting as for AWME3 samples. Then, 20 µL of AB was added to each well. AB specific activity was determined after 4 h of incubation at 37 °C with shaking at 160 rpm/min. The AB fluorescence measurement was performed at 540–570 nm of excitation and at 585–615 nm of emission. Data expressed as a percentage of viability, when compared with untreated cells. The MIC50 values were determined as the concentration of the AWME3 of *H. illucens* larvae fat that induced 50% inhibition of cell growth using the GraphPad Prism 7.0 software.

## Evaluation of *S. aureus* ATCC 55804 growth curves in the AWME3 presence

To validate the antimicrobial effect of AWME3 extract on the *S. aureus* ATCC 55804 growths, a turbidometric assay was used 14. Briefly, the assay was performed in 96-well microtiter plates with eight twofold serial of AWME3 dilutions in the range of 48–3000 µg/mL concentrations in Müller-Hinton broth. Likewise, the positive control (P/S) was subjected to the same procedure to obtain the final concentration of 0.6–78.1 μg/mL. Next, the plates were inoculated with bacterial suspensions at a final density of 5 × 10^5^ CFU/mL, all microplates were sealed and incubated at 37 °C for 24 h by shaking at 200 rpm/min. The OD600 values were recorded every 2 h intervals during 24 h. All tests were performed in triplicate, and each experiment was repeated three times.

### Salt tolerance assay

The effect of salt concentration on the bacterial cells viability in the presence or absence (for negative control) of AWME3 extract was conducted in a 96-well plate (TPP, Switzerland). The salt tolerance effect of *S. aureus* ATCC 55804 treated with the MIC and 2 MIC concentrations of AWME3 was evaluated on nutrient agar (NA) plates supplemented with different concentrations of NaCl. Fresh bacterial cultures were incubated at 37 °C overnight. After incubation, the overnight cultures of *S. aureus* ATCC 55804 were treated with AWME3, then incubated for 60 min at 37 °C. Then, the samples were serially diluted and inoculated on NA plates, supplemented with different concentrations of NaCl (0%, 2.5%, 5%, and 10%). (NA-NaCL plates). A bacterial culture without AWME3 was used as the control for each NA-NaCl plate. The results were expressed in terms of Log10 CFU/mL as the mean of three independent experiments.

### Scanning electron microscopy (SEM)

The scanning electron microscopy (SEM) was performed using a single colony of *S. aureus* ATCC 55804. A single colony was cultured in 5 mL of LB broth and incubated at 37 °C by shaking at 180 rpm/min overnight. The bacterial culture was centrifuged at 4000 × g for 10 min, and then bacterial pellets were washed 4 times by PBS (10 mM, pH 7.2) and cooled to 4 °C. The overnight culture of *S. aureus* ATCC 55804 was treated with 1-, 2-, and 4 MIC of AWME3. Likewise, the untreated samples (controls) were prepared and incubated in the same way. All bacterial cultures were harvested by centrifugation at 5000 × g at 4 °C for 10 min, then bacterial pellets were washed 3 times by PBS (10 mM, pH 7.2), and then 100 µL of every culture was spread on a glass slide (Thermo Fisher Scientific, Waltham, USA), dried under a sterile condition for 20 min. Subsequently, the bacterial cells were fixed overnight with 500 µL of 2.5% (v/v) glutaraldehyde at 4 °C. The fixed samples were washed twice with 10 mM PBS followed by dehydration in gradient ethanol solutions (50, 70, 90, and 95%) for 10 min. Finally, samples were viewed with SEM (TESCAN Co., Brno-Kohoutovic, Czech Republic).

### Transmission electron microscopy (TEM)

All samples of *S. aureus* ATCC 55804 were prepared by the same method described in SEM microscopy setting, however 0.5 mL of 2.5% (v/v) glutaraldehyde were added to the samples in 15 mL tubes and incubated overnight at 4 °C for fixation. After overnight incubation, the bacterial cells were fixed with 2% osmium tetroxide for 70 min after removing the 0.5 mL glutaraldehyde. The fixed samples were washed three times with PBS (pH 7.4). Then, the samples were dehydrated for 10 min in the mixture (1:1, v/v) of absolute ethanol and absolute acetone. The samples were transferred to a mixture (1:1, v/v) of absolute acetone and epoxy resin for 30 min followed by incubation 1.0 mL of pure epoxy resin overnight at a constant temperature 4 °C. Finally, the bacterial specimens were sectioned using an ultra-microtome (JEOL Ltd, Akishima, Tokyo, Japan). 5 µL of each culture aliquot was placed on the surface of a 300-mesh copper grid (Sigma-Aldrich, USA) and incubated at room temperature for 3 min. The excess fluid was removed, and bacteria were stained for 30 s with 1% uranyl acetate (Sigma-Aldrich, USA). Stained cells were imaged using transmission electron microscopy (TEM) (JEOL Ltd, Akishima, Tokyo, Japan), and at 10 000 × magnification.

### Statistical analysis

Statistical analysis was performed by two-way analysis of variance of repeated measurements (Two-way RM-ANOVA) to determine the statistically significant differences between treatments of the AWME3 susceptibility against tested bacteria strains compared to positive control. Differences between means were compared by Dunnett’s post-hoc test. Results of fatty acid profiles were analysed by ordinary one-way ANOVA, and Tukey’s post-hoc test was conducted to determine significant differences between treatment means. All the data were assessed by the standard deviation (SD) using the software GraphPad Prism 7 (GraphPad Software Inc., San Diego, CA, United States). The confidence level of statistical significance was adapted for 95% (p < 0.05).

## Results

### Determination of AWME3 chemical composition

The chemical composition of AWME3 was determined by GC–MS analytical method and included 33 compounds as was described in our previous publication15 and illustrate in Supplementary materials (Table S1 and Fig. S1). Relatively to the present study, we confirm that the major compounds detected in AWME3 were free fatty acids, and among them mainly the saturated fatty acids, (SFAs) and unsaturated fatty acids (USFAs) have been major. The SFAs included palmitic (C16:0, 21.76%), lauric (C12:0, 17.66%), stearic (C18:0, 5.82%), myristic (C14:0, 5.27%), arachidic (C20:0, 0.31%), and pentadecanoic (C15:0, 0.2%) acids. Among USFAs, cis-oleic acid (C18:1, 26.28%) was a major FA with palmitoleic (C16:1, 3.15%) and linoleic acid (C18:2, 0.21%) in addition, as shown in Table 1.

**Table 1:** Major fatty acids detected and quantified in AWME3 extract by GC–MS. Notes: RT, retention time.

	RT	Area %	Name of the compound	Molecular formula	Mol. weight (g/mol)	Similarity %
1	6.808	7.87	1,2,3-propanetriol (Glycerol)	C_3_H_8_O_3_	92	96
2	12.331	17.66	Dodecanoic acid (Lauric acid)	C_12_H_24_O_2_	200	97
3	14.058	5.27	Tetradecanoic acid (Myristic acid)	C_14_H_28_O_2_	228	97
4	14.753	0.2	Pentadecanoic acid	C_15_H_30_O_2_	242	91
5	15.286	3.15	cis-9-Hexadecenoic acid	C_16_H_30_O_2_	254	96
6	15.413	21.76	n-Hexadecanoic acid (Palmitic acid)	C_16_H_32_O_2_	256	96
7	16.35	26.28	Octadec-9-enoic acid (cis-oleic acid)	C_18_H_34_O_2_	282	95
8	16.439	5.82	Octadecanoic acid (Stearic acid)	C_18_H_36_O_2_	284	94
9	16.723	0.21	9,12-Octadecadienoic acid (Z,Z)- (Linoleic acid)	C_18_H_32_O_2_	280	91
10	17.345	0.31	Eicosanoic acid (Arachidic acid)	C_20_H_40_O_2_	312	90

### Antimicrobial susceptibility and MDR assessment of *Staphylococcus aureus* ATCC 55804 strain

**Fig. 1:**
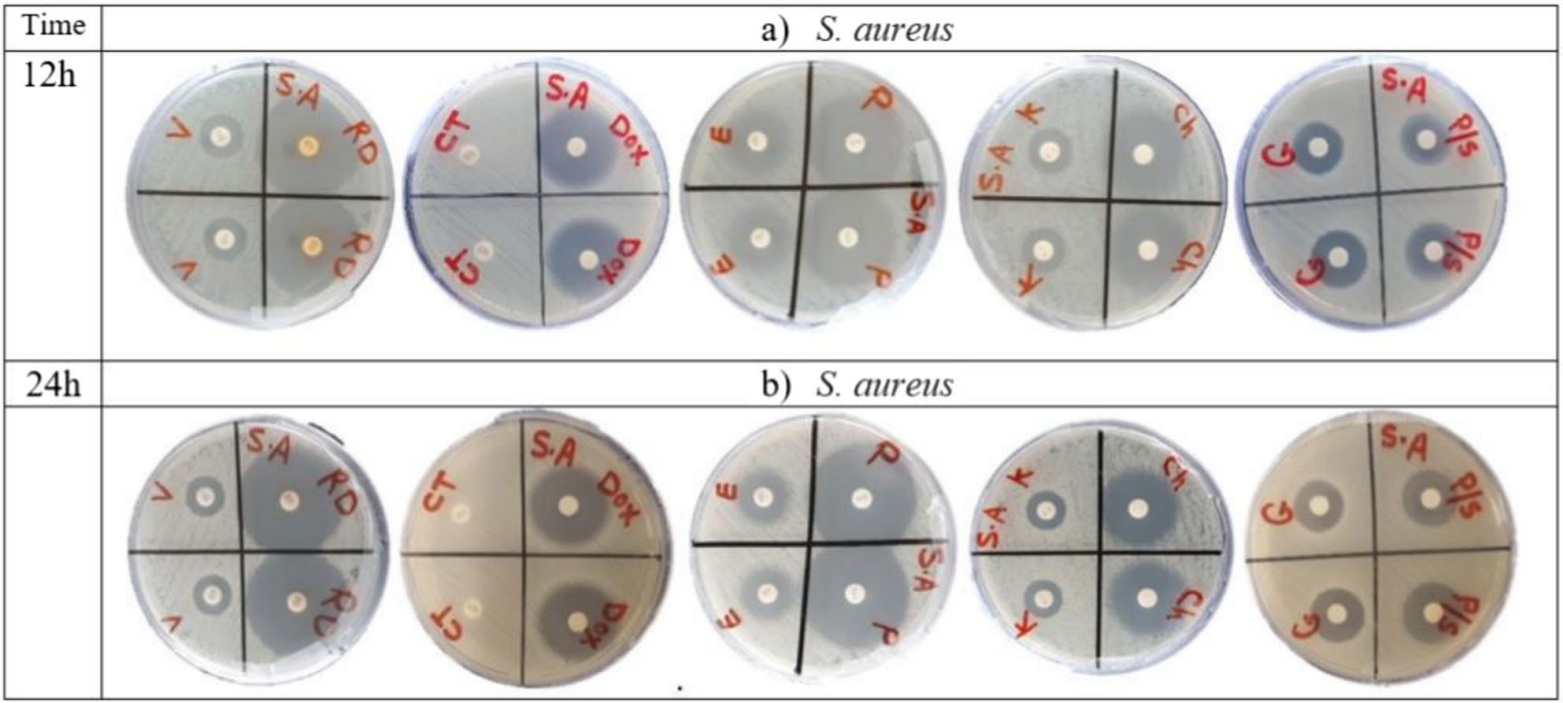
Susceptibility of *S. aureus* ATCC 55804 strain to eight groups of antibiotics confirmed by disk diffusion assay. All antibiotics were applied to the discs, following the protocol in the material and methods section, and gently transferred on the surface of the Muller Hinton agar Petri dishes with cultured bacteria strain at density 10^8^ (CFU/mL). All discs were applied in duplicates. The mean of the resulting IZD ± SD of three independent experiments was calculated and presented in Table 2.

The antibacterial susceptibility of ten antibiotics against Gram-positive *S. aureus* ATCC 55804 strain was determined by disc diffusion assay at 12 h and 24 h of incubation time. IZD values were interpreted according to CLSI guidelines breakpoints^23^ for *S. aureus* ATCC 55804. Our results showed that *S. aureus* ATCC 55804 strain was resistant to three (30%) of ten antibacterial agents, including kanamycin (K), colistin (CT), and vancomycin (VA) (Table 2, Fig. 1). *S. aureus* ATCC 55804 showed high susceptibility to rifampicin (RD), doxycycline (DOX), chloramphenicol (Ch) and penicillin (P). *S. aureus* ATCC 55804 demonstrated resistance throughout the entire 24 h of testing (Table 2).

**Table 2:** Antibiotic susceptibility of *S. aureus* ATCC 55804 strain.

Antibiotic	Potency	IZD (mm)				
		***S. aureus* ATCC 55804**		**Breakpoint (mm)**		
		12 h	24 h	R	I	S
G	10 μg	16.9 ± 0.68 (S)	16.5 ± 0.47 (S)	≤ 12	13–14	≥ 15
Ch	30 μg	25.9 ± 0.56 (S)	24.5 ± 0.44 (S)	≤ 12	13–17	≥ 18
K	30 μg	15.5 ± 0.45 (I)	13.5 ± 0.41 (R)	≤ 13	14–17	≥ 18
DOX	30 μg	21.6 ± 0.46 (S)	21.4 ± 0.43 (S)	< 12	13–15	≥ 16
P/S	10 U-10 μg	15.8 ± 0.5 (S)	14.5 ± 0.46 (S)	≤ 11	12–14	14–22
CT	10 μg	0 (R)	0 (R)	≤ 10	-	≥ 11
RD	5 μg	36.2 ± 0.83 (S)	35.5 ± 0.78 (S)	≤ 16	17–19	≥ 20
E	15 μg	17.5 ± 0.45 (I)	15.3 ± 0.87 (I)	≤ 13	14–22	≥ 23
VA	30 μg	14.4 ± 0.43 (R)	13.5 ± 0.87 (R)	-	-	≥ 15
P	2 U	34.75 ± 0.99 (S)	34.3 ± 0.82 (S)	≤ 28	-	≥ 29

Notes: G, gentamicin; Ch, chloramphenicol; K, kanamycin; Dox, doxycycline; P/S, penicillin–streptomycin; CT, colistin; RD, rifampicin; E, erythromycin; VA, vancomycin; P, penicillin. R, resistant; I, intermediate; S, susceptible; (0), means no inhibition zone around the disc on the plate; (-), not determined. Each value is a mean of three biological replicates.

### Antibacterial activity of AWME3 against MDR *S. aureus* ATCC 55804 strain

**Fig. 2:**
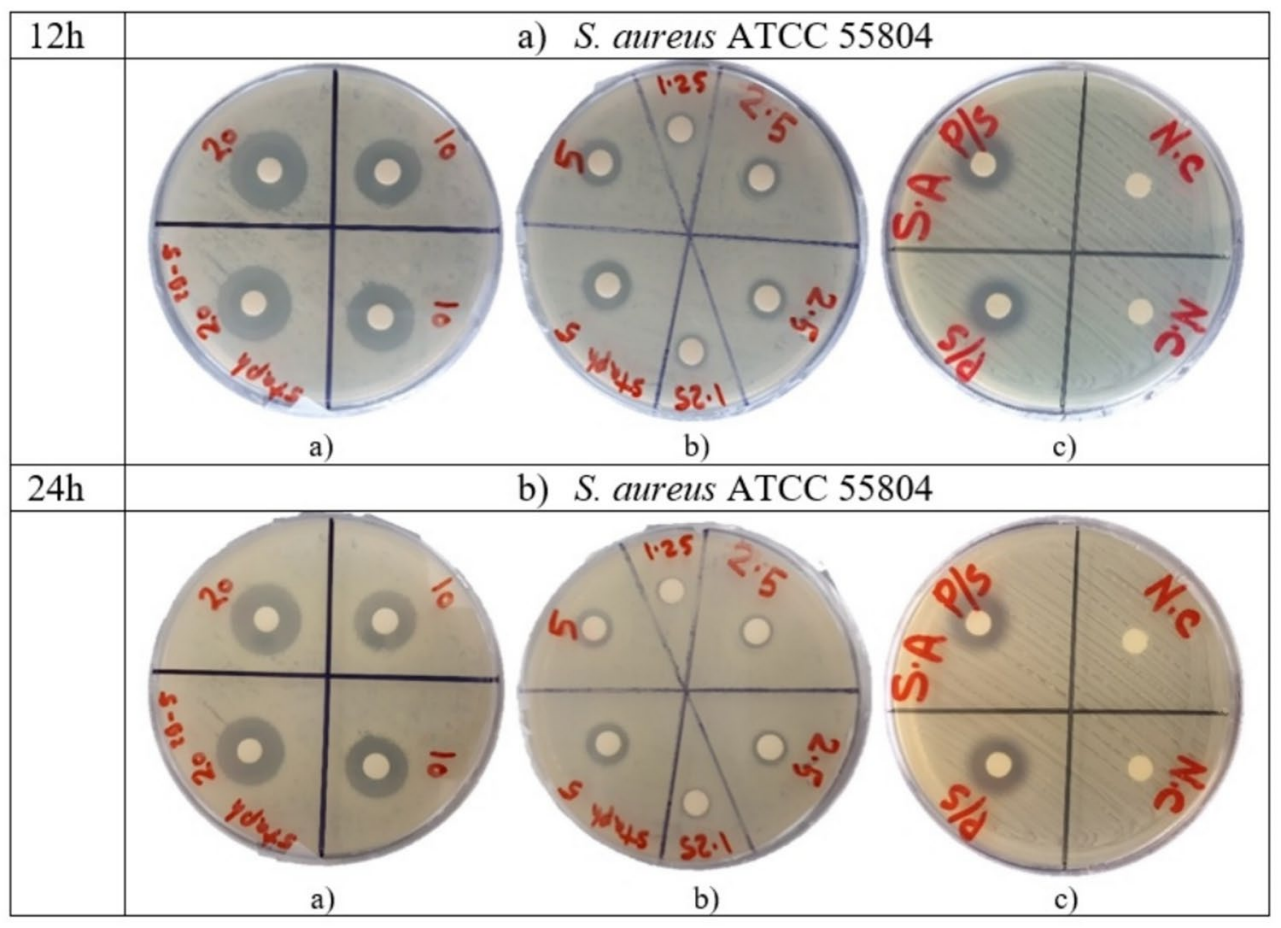
The disk diffusion assay of AWME3 against pathogenic bacteria strain.

Disc diffusion assay was to validate the antimicrobial susceptibility of AWME3 extracted from the same biomass of HI fat against MDR *S. aureus* ATCC 55804 strain. Bacterial cultures at optical density 10^8^ CFU/mL subjected to various 1.25, 2.5, 5, 10, and 20 mg/mL concentrations of the larval AWME3 extractME3, inhibition zone diameter (IZD) was determined by measuring the size of bacterial growth inhibition induced by AWME3. All measurements were recorded at 12 h and 24 h of incubation time (Table 3, Fig. 2).

**Table 3:** Inhibition zone diameters caused by AWME3 of *H. illucens* larvae fat against human pathogenic strains of *S. aureus* ATCC 55804.

Concentration (mg/mL)	*S. aureus* ATCC 55804	
	12 h	24 h
20	19.6 ± 0.79	18.4 ± 0.35
10	16.6 ± 0.51	15.5 ± 0.48
5	12.7 ± 0.46	11.4 ± 0.39
2.5	9.8 ± 0.29	8.6 ± 0.43
1.25	8.7 ± 0.42	7.6 ± 0.34
Positive control (P/S)	15.8 ± 0.5	14.5 ± 0.46
Negative control (N.ct)	ND	ND

Notes: ND, Not determined; N. CT, Negative control; P/S, Penicillin–streptomycin used as a positive control with a concentration (200 U/mL-200 µg/mL); each value is a mean of three biological replicates.

**Fig. 3:**
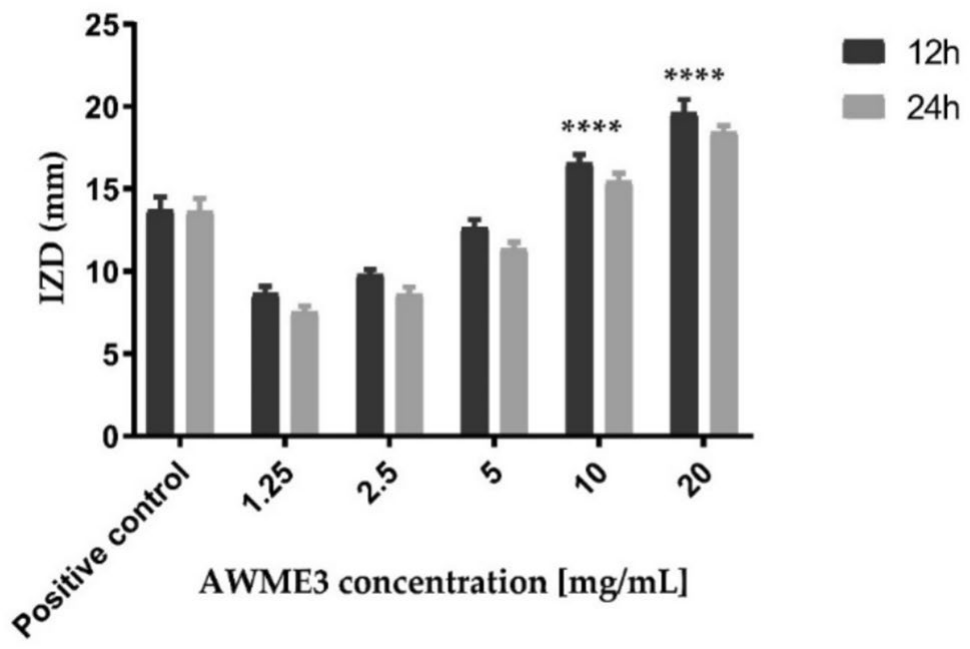
Antimicrobial sensitivity of AWME3 against *S. aureus* ATCC 55804.

Statistical analyses were conducted using two-way ANOVA to analyse our results and compare to the positive control (P/S). Results showed that a great significant difference occurred between the mean IZ sizes obtained by treating the human pathogenic bacteria strains with the AWME3 when compared to the IZ sizes caused by the positive control (P/S) (p ≤ 0.05). The highest inhibition zone sizes caused by AWME3 were 18.4 ± 0.35 mm against *S. aureus* ATCC 55804, when treated with 20 mg/mL at 24 h of incubation, compared to the positive control (P/S), which recorded 14.5 ± 0.46. ANOVA results showed a great significant (****p < 0.0001) IZD was recorded against *S. aureus* ATCC 55804, when they were subjected to a concentration of 20 mg/mL of AWME3 extract, respectively compared to the reference control at 12 h, and 24 h of incubation time (Fig. 2). Of note, bacterial resistance increased by the time for all tested strains proved by the decreased IZD value when bacteria strains were incubated for 24 h (Figs. 2, 3). Our results show that AWME3 has high potency against MDR bacteria, including *S. aureus* ATCC 55804 when they were treated with 10 and 20 mg/mL of AWME3. All MDR strains of *S. aureus* ATCC 55804 were inhibited at dose-dependent manner; moreover, all tested bacteria strains were eradicated and killed at 20 mg/mL concentration of AWME3.

### Bactericidal AWME3 activity against MDR human pathogenic bacteria strain

Microdilution assay was used to determine the MICs of all tested bacteria strains. As demonstrated in Table 4 the MICs of the AWME3 extract were determined against tested bacteria and all data was recorded after 24 h of incubation.

**Table 4:** Evaluation of the bactericidal and bacteriostatic activity of the AWME3.

Antimicrobial agent	*S. aureus* ATCC 55804		
	MIC (μg/mL)	MBC (μg/mL)	MBC/MIC
AWME3	190	380	2
Penicillin – streptomycin (P/S)	19.53	19.53	1

The cultural growth of tested bacteria strains was inhibited by AWME3 and the MICs recorded 190 µg/mL against *S. aureus* ATCC 55804. Tested strain were treated with different concentrations of the positive control in the range of 0.6–78.125 µg/mL. The minimum bactericidal concentration (MBC) was identified as the lowest concentration, which can kill 99.99% of the tested bacteria strains after 48 h of incubation time. The MBCs of AWME3 recorded 380 µg/mL against *S. aureus* ATCC 55804; likewise, the MBCs of positive control recorded 19.53 µg/mL. These results demonstrate that AWME3 has high activity against MDR bacteria strains. Bactericidal activity was determined based on the ratio of MBC/MIC. The ratio of MBC/MIC of AWME3 recorded two and one against *S. aureus* ATCC 55804 when they were treated with various concentrations of AWME3 (0.38–3.0 mg/mL). On the other hand, the ratio of MBC/MIC value recorded 1.0 and 2.0 of the positive control (P/S) against S*. aureus* ATCC 55804 (Table 4), when bacteria strain subjected to different concentrations with a range (0.6–78.125 µg/mL) of the reference antibiotic (P/S). Based on these results, AWME3 extract from HI larvae fat acted as bactericidal on Gram-positive bacteria *S. aureus* ATCC 55804.

### Effect of AWME3 on bacterial growth curves

**Fig. 4:**
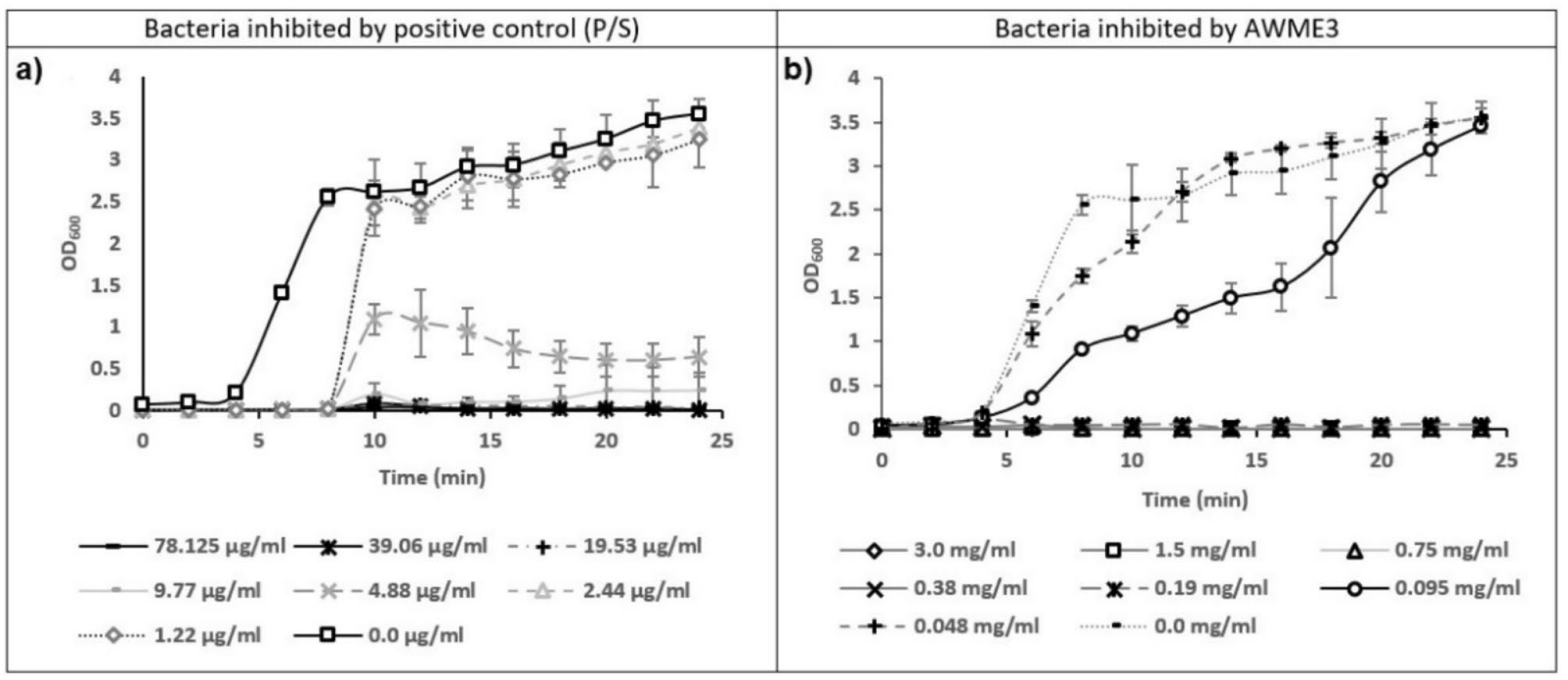
The growth curves of *S. aureus* ATCC 55804 treated with different concentrations of either AWME3 or P/S. Each data point is the average of three independent assays ± standard deviation of the mean (SD) and *p < 0.05 was significant.

Growth kinetics at OD_600_ used to study the growth curves for *S. aureus* ATCC 55804 as Gram-positive. The growth curves of *S. aureus* ATCC 55804 treated with different concentrations of the AWME3 extract of BSFL fat are shown in Fig. 4.

In the presence of 0.048 and 0.095 mg/mL of AWME3 extract, the growth curves of *S. aureus* ATCC 55804 included three phases: lag, exponential, and stationary phases. Since the bacterial culture density, including live and dead cells, was assessed by OD_600_, the decline phase could not be detected on the growth curves of *S. aureus* ATCC 55804 strain. The MBC values of the extract induced the inhibition of growth, which appeared as a straight line, parallel to the negative control. Untreated *S. aureus* ATCC 55804 reached the exponential growth phase rapidly, after about 4 h of incubation. The concentration 0.048 mg/mL of AWME3 reduced the period time of the log phase of *S. aureus* ATCC 55804 cells to begin at 4 h and stop at 16 h, compared to the untreated cells, which their log phase ended at 22 h (Fig. 4). When *S. aureus* ATCC 55804 cells were exposed to 0.095 mg/mL of AWME3, the log phase lasted at 24 h before the stationary phase began compared to the untreated *S. aureus* ATCC 55804 cells, which their stationary phase began early at 22 h. In addition, the log phase of treated bacterial cells of *S. aureus* ATCC 55804 recorded OD_600_ = 1.62 ± 0.27 unit at 16 h, compared to the negative control of *S. aureus* ATCC 55804, which recorded 2.94 ± 0.26 unit at the same incubation time. The concentration 0.38 mg/mL of AWME3 was previously determined as bactericidal and the bacterial growth phases were not detected on the growth curve when this concentration was used. Positive control (P/S) delayed the log phase of *S. aureus* ATCC 55804 when it was treated with different concentrations (1.22–9.77 µg/mL) since the log phase began at 8 h compared to the untreated bacteria which started early at 4 h. The most interesting that the concentration 4.88 µg/mL of the reference antibiotic stopped the log phase of *S. aureus* ATCC 55804 cells at 10 h and increased the decline phase to continue from 10 to 24 h. In addition, the concentration 9.77 µg/mL of P/S decreased the period time of the log phase to be 2 h and increased the stationary phase time to begin at 8 h, and continue until 24 h.

### Elucidation of bacterial cell viability via MIC50 assessment

**Fig. 5:**
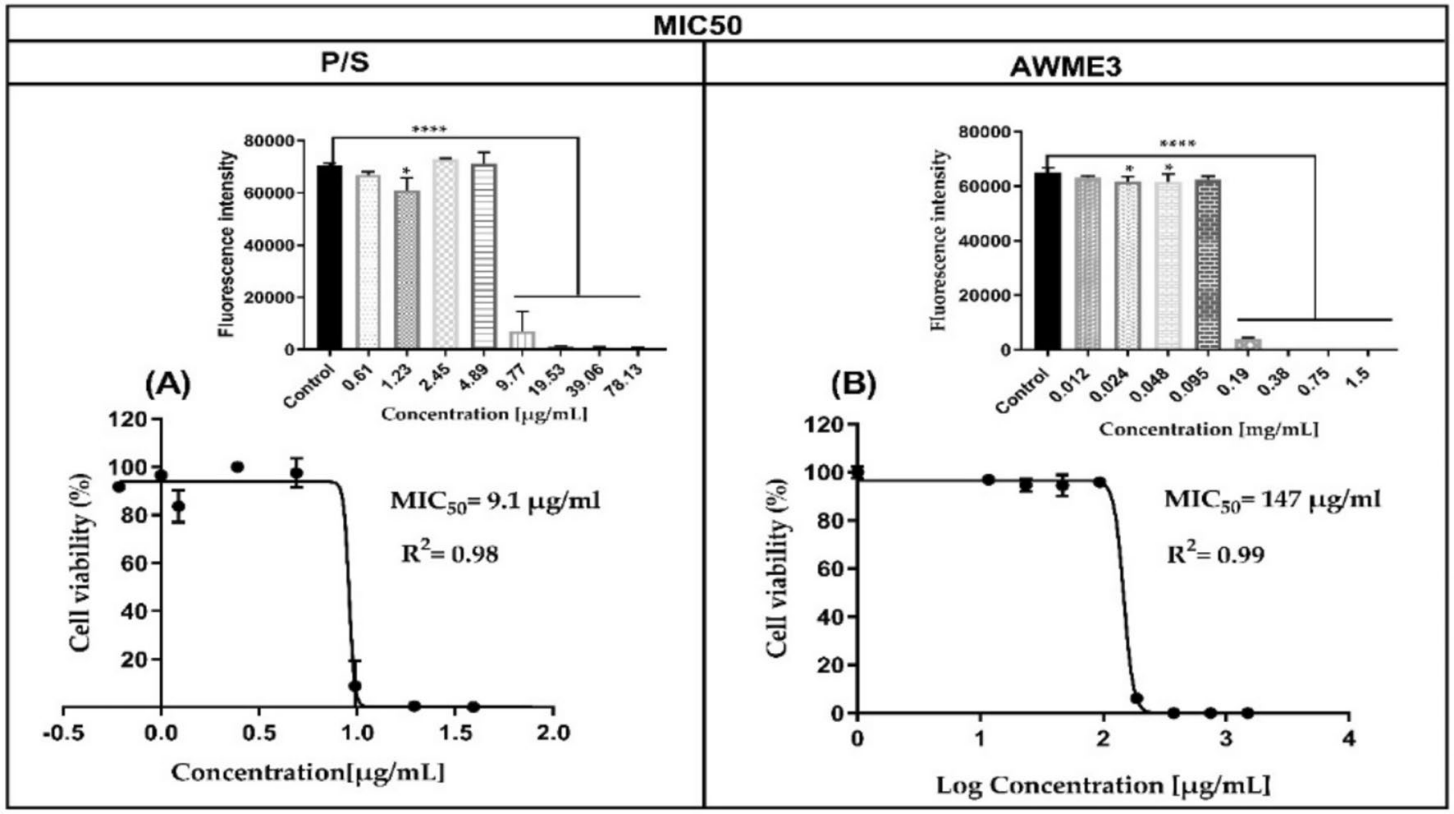
Effect of AWME3 from HI larvae fat on human pathogenic *S. aureus* ATCC 55804. Cell viability (%) was determined using the Alamar blue method. Error bars indicate the standard deviation of % viability obtained from three separated experiments, one-way ordinary ANOVA Dunnett’s multiple comparisons test (****p < 0.0001) was significant. The MIC50 values were calculated based on the reduction of the Alamar blue assay and compared to the positive control (P/S). The pathogenic cells viability was assessed after 24 h of incubation. (**A**) treatment with P/S; (**B**) treatment with AWME3. The MIC50 values were calculated using the non-linear regression mode of GraphPad Prism 7 (Graph Pad Software Inc., San Diego, CA, USA). The MIC50 values are the average of three independent experiments ± standard deviation error mean (SEM).

Cell viability test was performed using Alamar Blue (AB) assay. The data obtained from the AB assay demonstrated that 0.19, 0.38, 0.76, and 1.5 mg/mL of AWME3 have significant antimicrobial effects on planktonic forms of *S. aureus* ATCC 55804 (P < 0.05 compared with the control groups). AWME3 at the 0.38, 0.76, and 1.5 mg/mL concentrations inhibited and eradicated completely the growth of planktonic *S. aureus* ATCC 55804 cells over 100% after exposure to these concentrations, while 0.19 mg/mL showed inhibition of *S. aureus* ATCC 55804 planktonic cells with 95.9% compared to the untreated cells. Concerning planktonic *S. aureus* ATCC 55804 cells with inhibition rate (95.9–100%) showing a tendency of AWME3 dose-dependent manner (Fig. 5).

Our results highlighted that the concentration of AWME3 inhibited 50% of *S. aureus* ATCC 55804 (MIC50) was 147 μg/mL, when bacterial cells were subjected to various concentrations in the range 0.012–1.5 mg/mL of AWME3 concentrations. The MIC50 values of the positive control (P/S) recorded 9.1 and 6.07 μg/mL against *S. aureus* ATCC 55804, after exposure to different concentrations (0.6–78.125 μg/mL) of P/S at 24 h (Fig. 5). Our study demonstrated that the AWME3 extract of the *H. illucens* larvae fat exhibits high activity against MDR nosocomial bacteria strain.

### Salt tolerance effect

**Fig. 6:**
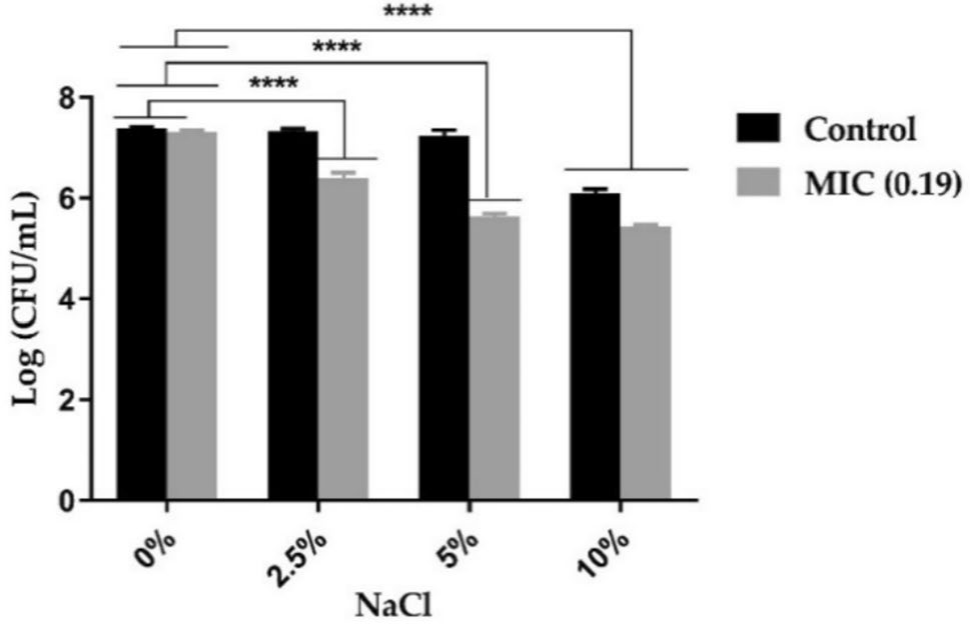
Effect of AWME3 extracted from *H. illucens* larvae fat at MIC and 2 MIC concentrations on the reduction of salt tolerance of *S. aureus* ATCC 55804. All values are represented as mean ± SD, in triplicate (n = 3). Data were analyzed by two-way ANOVA, followed by Dunnett’s multiple comparisons test. Data represented a significant difference as compared to the control without NaCl and p < 0.05 was significant.

Salt such as sodium chloride acts as a selective agent for bacteria, enhances changes in osmotic equilibrium balance, and interferes with membrane permeability. It is known that the high salt concentration inhibits different types of bacteria but allows salt-tolerant organisms to grow^24^. To examine whether the antibacterial activity of AWME3 was compromised and effective in the presence of salts, we treated *S. aureus* ATCC 55804 with different concentrations of AWME3 under various salt concentrations. The salt tolerance potential of *S. aureus* ATCC 55804 treated with AWME3 at the MIC and 2 MIC concentrations is shown in Fig. 6. The proportion of cells that were able to form colonies in NA-NaCl plates compared to NA plates (salt tolerance) was represented in Fig. 6. When the bacteria pre-treated with AWME3 and then inoculated on nutrient agar media supplemented with NaCl 0%, 2.5%, 5% and 10% of NaCl concentrations, a significant decrease in the number of colony-forming units at 10% of NaCl and the survivors colonies were 82.52% for *S. aureus* ATCC 55804, compared to the control without NaCl (****p < 0.0001) (Table 4, Fig. 6).

Neither the controls nor the treated samples showed any growth of bacteria in the NA plates supplemented with 10% NaCl for *S. aureus* ATCC 55804 treated with 2 MIC (Fig. 6). Pre-treated NA plates supplemented with 2.5% and 5% showed a non-significant decrease in survivor colonies compared to the control without NaCl for *S. aureus* ATCC 55804 (Fig. 6). All treated plates with 0.19 mg/mL of AWME3 showed high significant variance and the number of survivors colonies were sharply decreased (****p < 0.0001), except the plates of NA without NaCl and treated with MIC of AWME3 showed non-significant variance in the survivors colonies compared to the untreated control without NaCl of *S. aureus* ATCC 55804 (Fig. 6, Table 5).

**Table 5:** Salt tolerance of *S. aureus* ATCC 55804 treated with MIC and 2 MIC of AWME3 on NA plates with 0, 2.5, 5, and 10% NaCl.

Treatments	Log CFU/mL	Log CFU/mL	Log CFU/mL	Average	STD	Survivors
CT NA without NaCl	7.4	7.34	7.40	7.38	0.25	100
NA + 2.5%	7.30	7.32	7.38	7.33	0.20	99.37
NA + 5%	7.30	7.10	7.3	7.23	0.21	98.01
NA + 10%	6.17	6.00	6.10	6.09	0.10	82.52
NA + MIC	7.30	7.25	7.35	7.30	0.25	98.92
MIC + 2.5%	6.30	6.50	6.41	6.40	0.50	86.77
MIC + 5%	5.70	5.58	5.63	5.64	0.60	76.38
MIC + 10	5.47	5.43	5.39	5.43	0.25	73.58
CT NA + 2 MIC	0	0	0	0	0	0
2 MIC + 2.5%	0	0	0	0	0	0
2 MIC + 5%	0	0	0	0	0	0
2 MIC + 10%	0	0	0	0	0	0

Notes: CT, control; NA, nutrient agar; STD, standard deviation; each value is a mean of three biological replicates.

### Alteration in cell morphology of *S. aureus* ATCC 55804 treated with AWME3

Among the different microscopy techniques, scanning electron microscopy (SEM) is useful to characterize the cell surface morphology, while transmission electron microscopy (TEM) facilitates the characterization of surface morphology along with the density of inner cytoplasmic constituents^25^. To understand the mode of action of fatty acids and their derivatives in AWME3 from HI larvae fat, morphological alterations of *S. aureus* ATCC 55804 cells were observed using SEM. The bacterial cells were treated with different concentrations of AWME3 which are 0.5 MIC (Fig. 6b), MIC (Fig. 6c, d, e, f), and 2 MIC (Fig. 6g, h, i).

**Fig. 7:**
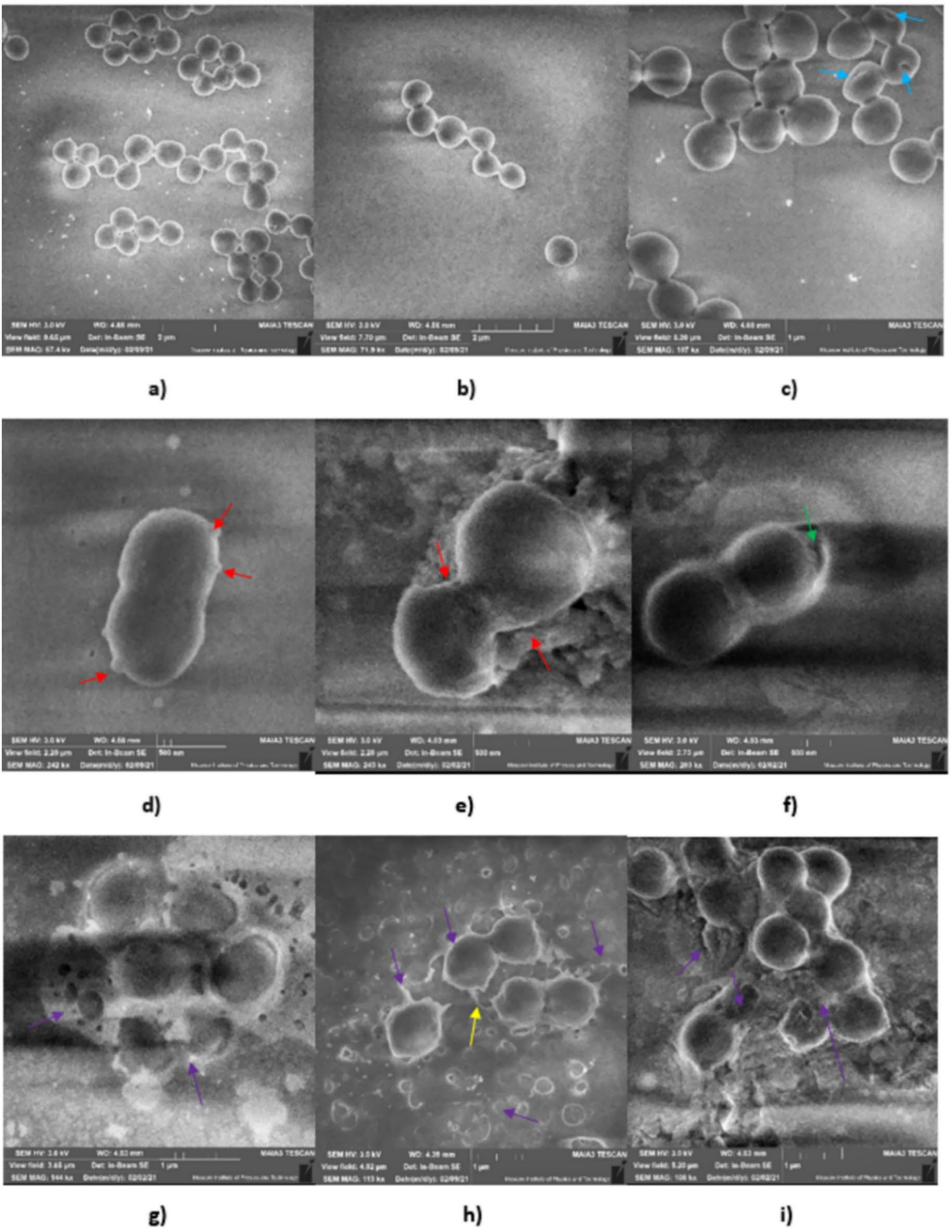
SEM images of *S. aureus* ATCC 55804 cells. (**a, b**) Untreated control; (**c**) Treated with MIC (0.19 mg/mL) for 12 h.; (**d, e, f, g**) Treated with 2 MIC (0.38 mg/mL) of AWME3 for 20 min; (**g, h, i**) Treated with 4 MIC (0.75 mg/mL) of AWME3 for 10 min. Blue arrows show small pores formed inside the cell membrane, red arrows indicate cell wall swelling and collapsing, green arrow refers to the vacuole or periplasmic space formation and cell wall deformation, violet arrows demonstrate lysed cells, cell wall ruptured completely, cell debris and yellow arrow shows blebs and loosed intracellular compartments.

SEM images of *S. aureus* ATCC 55804 cells were taken at 12 h for MIC, 20 min for 2 MIC, and 10 min for 4 MIC of AWME3 and incubated at 37 °C to study the possible mechanism(s) of action of AWME3 of HI larvae fat on bacterial cells. As shown in Fig. 7, the surfaces of the untreated cells of planktonic *S. aureus* ATCC 55804 were relatively smooth and continuous with good structural integrity, intact, and with a completely spherical shape (Fig. 7a, b). After the treatment of *S. aureus* ATCC 55804 cells with 0.5 MIC of AWME3, small pores were detected obviously, cells were still smooth, spherical, intact, and minor morphological changes occurred (Fig. 7c). Protrusions like those that buds formed on the cell surface, the membrane became corrugated and/or partially swelled whereas MIC (0.19 mg/mL) of AWME3 treatment for 12 h min caused severe morphological changes with late cell division (Fig. 7c).

The cell boundaries were faint and unclear when treated with the bactericidal 2 MIC (0.38 mg/mL) of AWME3 for 20 min, where the cells were collapsed, roughed, wrinkled, and deformed, furthermore, irregular shapes, gaps, and vacuoles were obvious after treatment (Fig. 7e, f, g). When the concentration of AWME3 reached 4 MIC (0.75 mg/mL), the *S. aureus* c ATCC 55804 cells appeared with indentations, blebs, collapsed, lysed, and non-integral cell morphology (Fig. 7g, h, i). Ghost cells appeared due to the loose of the cytoplasmic material. These results showed that AWME3 caused obvious destruction on the cell wall/membrane of *S. aureus* ATCC 55804*,* indicating that the components of cell walls/membranes of *S. aureus* ATCC 55804 have severe alterations with obvious changes. Based on these results AWME3 interacts directly with microbial cell walls/membranes to increase the membrane permeability and cause rapid cell death. A significant number of *S. aureus* ATCC 55804 cells failed to retain their oval shapes after treatment with 0.75 mg/mL of AWME3. The formation of ghost cells with distorted cell walls indicated the bactericidal action of the AWME3. These results suggested that interference with cell membrane permeability affected the viability of the cells. Based on these findings, this phenomenon suggests that the active substances from AWME3 from HI larvae fat may act on the cell membrane or extracellular proteins, generating small pores and leading to the destruction of bacterial cell growth.

### Alterations of cell compartments of *S. aureus* ATCC 55804 strain after exposure to AWME3

Electron microscopy is a popular measurement technique for investigating the morphological structure of bacterial cell samples and can be utilized to study the effects of antimicrobial lipid treatment^25^. Among the different techniques, transmission electron microscopy (TEM) facilitates the characterization of surface morphology along with the density of inner cytoplasmic constituents. To prove our hypothesis that AWME3 is considered an antibacterial agent, that disrupts the bacterial cell wall/cell membrane, and to confirm the damage of the *S. aureus* ATCC 55804 membranes, TEM micrographs were used to achieve our hypothesis.

**Fig. 8:**
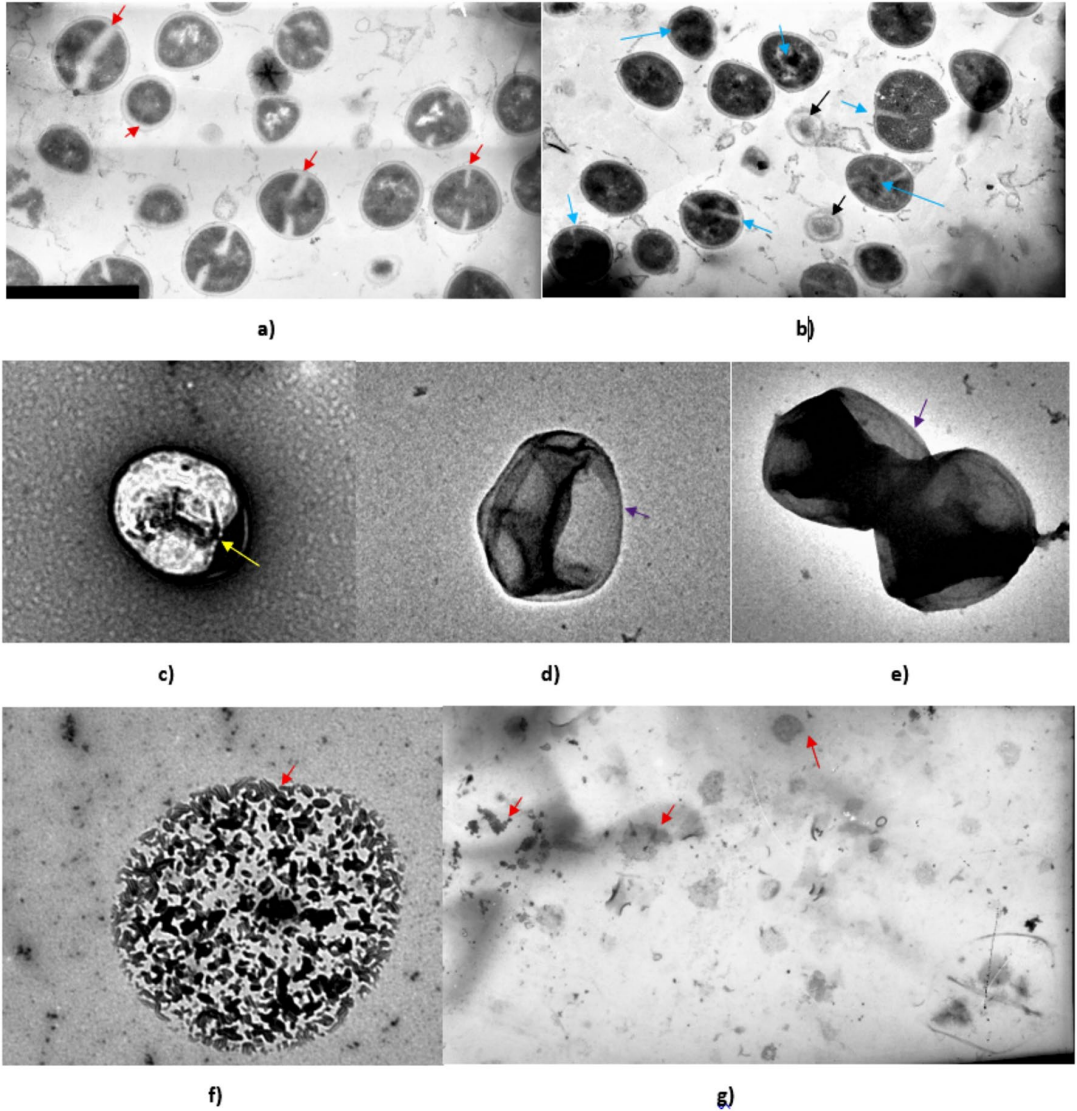
TEM micrographs of untreated and treated *S. aureus* ATCC 55804. Untreated cells looked round and intact, with a well-defined cell membrane and the red arrows indicate intact septa (**a**). The onset of septation (red arrows) and the cross-wall formation were noticed. After treatment for 12 h with the MIC (0.19 mg/mL) of the AWME3; (**b, c**) Cells appeared malformed with shrunken cytoplasmic material and aberrant septa; (**d, e**) Cells appeared with non-membrane-enclosed bodies (denoted by violet arrows) after treatment with 2 MIC (0.38 mg/mL) for 30 min. Some lysed cells were also noted (**f**), as well as null cells (**g**) were also noted after treatment with 4 MIC (0.75 mg/mL) for 10 min.

The membrane damage was obvious in Fig. 8, where the homogeneous cell walls with intact cytoplasm can be observed by TEM. Untreated cells of *S. aureus* ATCC 55804 grown under culture medium displayed the typical features of *Staphylococci* morphology: smooth rounded cells with an intact thick cell wall envelope and well-defined membranes (Fig. 8a).

The cytosol exhibited a homogeneous electron density and proliferating cells with a central septum (red arrows) were commonly observed. The cell wall showed a dipartite structure consisting of an outer layer that shows a highly stained fibrous surface and an electron-dense inner thin zone. Micrographs of untreated *S. aureus* ATCC 55804 cells showed well-defined septa formation occurring primarily through the middle of the cell, dividing the cell into two symmetrical daughter cells (Fig. 8a). Cells exhibited more diffuse septa and predominantly more “asymmetrical” cell divisions (blue arrows), treatment with MIC of AWME3 for 12 h affected cells, which displayed a rough surface and the electron density in the cytoplasm appeared to be dark, heterogeneous (Fig. 8b). Cells appeared abnormal with wrinkled walls, enlarged, and periplasmic spaces (black arrows) appeared in small cells with shrunken cytoplasm (Fig. 8b), compared to the untreated cells. Cytoplasmic material was condensed at the center of the cell with the presence of a large cavity (yellow arrow) at the anterior of the cell, the appearance of a clear zone, and retained the outer cell membrane (Fig. 8c). After treatment with 2 MIC of AWME3 for 20 min, cell wall and cell membrane appeared undistinguished, or loosed. In addition, dark condensed cytoplasmic compartments appeared heterogeneous, shrunken, and wrinkled cell walls (violet arrows) as shown in Fig. 8d, e. Exposure to a high concentration of 4 MIC (0.75 mg/mL) of AWME3 for 10 min led to complete degradation of the cell wall and cell membrane, dissociation of the intracellular content with rough surface (Fig. 8f). Moreover, *S. aureus* ATCC 55804 cells lysed completely, and cell debris were detected (Fig. 8g). The findings above further confirmed the disintegrating process of cell interior structure, which were consistent with the findings in SEM examination, and an obvious reaction happened between the AWME3 and cell wall membrane, which led to disintegration and cell death.

## Discussion

Antibiotic resistance for most common bacterial pathogens is considered a major concern in public health settings worldwide. Urgent need for exploring new natural and safe alternatives of broad‐spectrum antimicrobials to combat MDR pathogens, which threaten human and animal health. The antibiotic resistance profile of *S. aureus* ATCC 55804 was determined using the disk Diffusion Assay (Table 2). *S. aureus* ATCC 55804 was resistant to vancomycin (glycopeptide) and colistin (polymyxin E). Our findings declared that *S. aureus* ATCC 55804 was MDR-strain when subjected to ten commercial antibiotics belonging to eight groups (30%) (Table 2, Fig. 1). In addition, *S. aureus* ATCC 55804 strain was classified to be MDR bacteria^20,26^.

The antibacterial lipids isolated from insects have paid significant attention in recent years due to their potential to be used as natural alternatives to classic antibiotics. Lipids derived from insects, particularly those from HI larvae, have been found to exhibit antimicrobial properties against various bacterial strains, including Gram-positive and Gram-negative bacteria^16,27^.The insect-derived antimicrobial lipids have potential applications as natural feed additives to enhance animal health and as an alternative to antibiotics. Additionally, these lipids can be incorporated into personal care products and cosmetics, offering antibacterial and antiviral properties for human use. The antimicrobial lipids isolated from insects have shown significant activity against *S. aureus* whereas Bader and Mekhlif^28^ found that grubs of *Pentodon algerinum* extract, body extract of *Gryllotalpa gryllotalpa* and, *Gypsonoma euphraticana* larvae feces, showed promising antibacterial activity against *S. aureus*. Also, Ma^29^ stated that the shell of *Cryptotympana pustulata* Fabricius and the *Mole cricket* body exhibited significant antibacterial activity against *S. aureus*. Notably, the methanol extract of *Ailolopus thaiossinus* was more effective than the standard drugs (ceftriaxone) against *S. aureus*, and liposomal oleic acids showed great activity in eradicating drug-resistant *S. aureus*^30,31^.

These studies collectively suggest that insect-derived antibacterial lipids have the potential to combat *S. aureus* infections. The mechanisms of antimicrobial lipids against Gram-positive bacteria involve interactions with bacterial membranes, disruption of essential cellular processes, and induction of structural damage, ultimately leading to bacterial death. Interestingly, the *S. aureus* strain developed novel resistance strategies to inhibit the effects of antibacterial FAs through detoxifying FAs by esterifying them with cholesterol using the enzyme Lip2^32^. Understanding these mechanisms is crucial for developing novel lipid-based antimicrobial agents with enhanced efficacy and specificity to combat the novel resistance induced by MDR bacteria.

The primary results of the investigation activity demonstrated that the AWME3 extract showed marked antibacterial activity with IZD of 18.1 and 18.4 mm (Table 2 and Fig. 2). Our results stated that AWME3 extracted from HI larvae fat exhibited high efficacy compared to these extracts isolated from *Forcipomyia nigra* or HI larvae^10,33^.

AWME3 was extracted sequentially from the same biomass (3 g) of *H. illucens* larvae fat, then the thrice extract AWME3 was the most potent extract among other extracts, furthermore AWME3 was investigated and analysed using GC–MS (Table 1). The most abundant unsaturated fatty acids (UFAs) were Cis-oleic and palmitoleic acids, while saturated FAs (SFAs) were palmitic, lauric, stearic, and myristic acids^34^. Therefore, the antibacterial properties against the selected MDR microorganisms of AWME3 induced by FAs constituents activity demonstrated high effectiveness compared to other literature^35,36^. The most interesting was that our AWME3 showed significant activity and stability at high temperature (50 °C) (unpublished data), compared to commercial fatty acids and glycerides, which demonstrated low activity at high temperature 23 °C against *L. monocytogens*^37^*.* Recently, AWME3 was identified as a promising antibacterial agent, which showed remarkable antibacterial activity against various pathogenic bacteria, including the clinical pathogens *K. pneumonia spp* and *S. aureus*^13,15^*.* Furthermore, the activity of AWME3 isolated from BSFL fat was more effective than the activity of larvae oil^15^. Taken together, the antimicrobial properties of AWME3 including MIC (0.19–0.38 mg/mL), MBC (0.38 mg/mL), and MIC50 (0.147–0.222 mg/mL) have significant inhibition and eradication of both Gram-negative and Gram-positive bacteria, compared to the data of other literature^27,38,39^. Our findings promote the capacity of AWME3 as a new therapeutic candidate with significant activity at dose-dependent manner against bacteria growth curves (Fig. 3). Additionally, Herndon et al^40^ documented that FAs have a significant effect on the growth curves of Gram-negative bacteria, and these results highlight the potential advantage of using AWME3 to treat severe bacterial infection. The most interesting is that AWME3 is effective in the presence of different concentrations of NaCl and exhibited a significant reduction in the survival colonies, and these results were in line with previous studies^41,42^.

FAs are identified as non-traditional agents, which target multiple mechanisms that enhance bacterial resistance to antibiotics or provoke virulence development instead of killing bacteria directly. Nowadays, the development of new therapeutic agents is needed to target such mechanisms, where these agents could decline antibiotic use, and this could lead to reducing the evolution of persistence and resistance mechanisms generated via various bacteria species^43^. Fatty acids are commonly used by the food industry to inhibit bacterial growth^44^. The antibacterial activity of fatty acids has been proposed to be influenced by hydrophilic and lipophilic characteristics, which enable incorporation into the bacterial cell membrane^45,46^. A recent study by Schlievert and Peterson^47^, reported that fatty acids glycerides were more potent than their source, such as glycerol monolaurate (GML) and lauric acid (LA). Furthermore, LA converted to GML can destroy bacterial lipid membranes^48^. A recent study showed that the activity of SFAs increased sharply when lowering the pH, where a decrease in the pH to only 0.5 units could change the state from non-lethal to lethal conditions. On the other hands, the bactericidal activity of GLM was not pH-dependent, thus GML is considered more environmentally stable and can be used in crop protection.

Obvious changes in the cell morphology of *S. aureus* ATCC 55804 strains were detected by scanning electron microscopy analysis. Our findings showed that *S. aureus* ATCC 55804 treated with different concentrations (0.19–0.76 mg/mL) of AWME3 exhibited surface roughness, irregular cell shapes and a high degree of collapse and cell lysis. These results can be elucidated by assuming that the mode of AWME3 action led to cell membrane damage. Moreover, the AWME3 promotes the cytoplasmic leakage of *S. aureus* ATCC 55804 strain (Figs. 6g, Fig. 7h), implicating that FAs surfactants of AWME3 can react with bacterial membrane or extracellular protein leading to increase the membrane permeability^13^. The prime target of FAs is known to be the cell membrane antimicrobial substances, which enter into the cytoplasm, thereby causing bacterial death. Particularly, hydrogen ion is considered a robust bacterial killing agent in the cytoplasm, but it cannot pass the cell through an intact cell membrane owing to its polarity. However, membrane disruption can occur directly and rapidly due to hydrogen ions uptake from the extracellular environment.

The cell membrane prominence counts on many essential functions, making it one of the principal targets for developing new antibacterial agents. Sun and his collaborators stated that UFA such as myristoleic acid, palmitoleic acid, α-linolenic acid, and oleic acid with one or more double bonds have the ability to provoke membrane disruption in bacteria^49^. Investigation via scanning electron microscopy (SEM), shows that these UFAs could completely disrupt the cell membrane of gram-negative or gram-positive bacteria^50^. Additionally, morphological changes, including much rougher bacterial membranes, have been observed in *S. mutans* treated with docosahexaenoic acid, and eicosapentaenoic acid^51^.Moreover, Le and Debois reported that eicosapentaenoic acid displayed disruptive membrane activity against *B. cereus* and *S. aureus*^52^. They quantified the leakage of 260-nm absorbing material, including genetic material, from the bacterial cells in suspension and showed an increase of absorbing material in the extracellular space as the concentration of eicosapentaenoic acid increased, suggesting membrane disruption.

Studies also show that FAs diffuse through the cell membrane causing transient or permanent pores (Fig. 6c, Fig. 7c), leading to loose membrane permeability and cell death. The expected mechanisms underlying the bactericidal activity-mediated FFAs have been widely investigated. Dissociated FFAs penetrate the cell membrane and enter inside the cytoplasm, where they discharge into charged anions and protons, where they altering the equilibrium of hydrogen ions inside the cell, and increase the pH value^17^. The presence of charged ions and protons inside the cytoplasm can alter the intracellular pH homeostasis, leading to blocking the major metabolic reactions and generating toxic anions accumulation^17,53,54^.

Lauric acid (LA) displaying disruptive membrane properties, where Yang et al.^55^ detected the effect of LA on the integrity of the cytoplasmic membrane of *C. difficile*. In this study, *C. difficile* released nucleic acids with high concentration compared to the untreated control when treated with LA. Furthermore, LA was reported to disturb the bacterial membrane integrity. These results were confirmed by ultrathin-section transmission electron microscopy (TEM) when abnormal cell morphology and cytoplasmic content leakage were observed. Similarly, LA caused alteration in the cell morphology of *S. aureus* and MRSA by inducing a tubular formation on the lipid bilayer resulting in cell lysis^56^. Cis-6-hexadecenoic acid also was explored to cause an inhibitory effect or can kill *S. aureus* even at low concentrations^57^. In addition, antibacterial mechanisms of the cis-6-hexadecenoic were demonstrated to cause losing of membrane integrity by disrupting the proton motive force, increasing membrane fluidity, and electron transfer pathways^57^. Noteworthy, liposomal α-linolenic acid mechanism was documented that it may disrupt the plasma membrane leading to separation of the outer membrane away plasma membrane leading to loss of cytoplasmic contents.

TEM analysis visualized obvious changes that occurred within the bacterial cell membrane and intracellular compartments of *S. aureus* ATCC 55804 after treatment with different concentrations (0.19–0.75 mg/mL) of AWME3 (Fig. 8). Severe alteration in the cell wall, cell membrane and cytoplasmic contents (Fig. 8 b-g), where cells appear with asymmetrical septa, without intact cell wall and ruptured membrane. This study paved the way for future studies, which aim to perform comparative analyses with purified standard fatty acids such as oleic acid or palmitoleic acid compared with AWME extract for further delineate the contribution of specific fatty acids in fighting MDR bacterial pathogens.

## Conclusion

In conclusion, our research indicated for the first time the antibacterial activity and mechanism of antimicrobial action of AWME3 on MDR nosocomial *S. aureus* ATCC 55804 strain. Our presented results clearly demonstrate that the combination of SFAs and USFAs in AWME3 isolated from *Hermetia illucens* fly larvae fat was able to eradicate *S. aureus* that causes a global public health problem. Our discovery elucidates that newly developed AWME3 antimicrobial of natural origin may be a promising alternative candidate in the fight against the overuse of the traditional antibiotics, and to stop spread of MDR nosocomial pathogens. We elucidated the mechanism of bactericidal action of AWME3 FAs due to severe morphological changes in the bacterial cell membrane, including destruction of cell compartments, loss of membrane integrity leading loss of the cytoplasmic contents and cell death by applying SEM and TEM microscopy approaches. The SEM and TEM observations confirm the hypothesis that FAs in AWME3 may react with the cell membrane or extracellular proteins, generating small pores and leading to increasing cell membrane permeability, losing electrolytes, and cytoplasmic materials followed by disintegration and bacterial cell death. The phenomenon of salt tolerance proved the stability of AWME3 extract, which has activity at high concentrations of salt. Thus, the AWME3 extract from BSFL fat may provide a promising antimicrobial agent for therapeutic applications against nosocomial drug-resistant bacteria.

## Supplementary Information

Supplementary Information.

## Data Availability

All data generated or analyzed during this study are included in this article. All bactericidal efficacy of AWME3 data, including MBC/MIC against MDR S aureus ATCC 55804 strain were generated by this article authors. Image data were extracted from SEM and TEM microscopy study of mechanism of antibacterial AWME3 action. Additional information and data are available from corresponding authors upon request.

## Declarations

### Human and animal resources

The authors state that no humans or animals were involved in the study.
