# Supplementary material for: Mechanism of bactericidal efficacy against nosocomial pathogenic Staphylococcus aureus strain caused by fatty acids from Hermetia illucens larvae fat
Source: Sci Rep. 2025 Aug 19;15:30305. doi: 10.1038/s41598-025-15858-0 (PMC12365018; doi:10.1038/s41598-025-15858-0)
Supplement: Supplementary file 1 — Supplementary Information. [file 41598_2025_15858_MOESM1_ESM.pdf]

Mechanism of bactericidal efficacy against nosocomial pathogenic *Staphylococcus aureus* strain caused by fatty acids from *Hermetia illucens* larvae fat

Heakal Mohamed [a, b*ƚ], Elena Marusich [b*ƚ], Margarita Pustovalova [b], and Sergey Leonov [b, c]

[a] Agricultural Research Center (ARC), Plant Protection Research Institute (PPRI), Dokki, Giza, Egypt

[b] Institute of Future Biophysics, 141701 Dolgoprudny, Moscow Region, Russian Federation

[c] Institute of Cell Biophysics, Russian Academy of Sciences, 142290 Pushchino, Moscow Region, Russian Federation

ƚ Equally contributed in the work

*** Correspondence:**

Corresponding authors:

Elena Marusich (marusich.ei@mipt.ru)

Heakal Mohamed (m.heakal@phyestech.edu)

S. No	RT	Area %	Name of the compound	Molecular formula	Mol. weight (g/mol)	Similarity %
1	3.798	0.11	2-Propanone, 1-hydroxy-	$C_3H_6O_2$	74	83
2	5.928	0.85	1,2-Propanediol, 3-chloro- (Glycerol .alpha.-chlorohydrin)	$C_3H_7ClO_2$	110	94
3	6.808	7.87	1,2,3-propanetriol (Glycerol)	$C_3H_8O_3$	92	96
4	10.403	0.3	n-Decanoic acid (Capric acid)	$C_{10}H_{20}O_2$	172	84
5	11.552	0.11	Benzene, octyl-	$C_{14}H_{22}$	190	82
6	11.84	0.14	Beta.-D-Glucopyranose, 1,6-anhydro- (Levoglucosan)	$C_6H_{10}O_5$	162	77
7	12.331	17.66	Dodecanoic acid (Lauric acid)	$C_{12}H_{24}O_2$	200	97
8	13.923	0.13	2,4-Dodecadienal, (E,E)-	$C_{12}H_{20}O$	180	80
9	14.058	5.27	Tetradecanoic acid (Myristic acid)	$C_{14}H_{28}O_2$	228	97
10	14.683	0.27	Dodecanoic acid, ethenyl ester	$C_{14}H_{26}O_2$	226	77

11	14.753	0.2	Pentadecanoic acid	$C_{15}H_{30}O_2$	242	91
12	14.936	0.64	3-Cyclopentylpropionic acid, 2-dimethylaminoethyl ester	$C_{12}H_{23}NO_2$	213	92
13	15.187	0.23	Hexadecanoic acid, methyl ester (Palmitic acid methyl ester)	$C_{17}H_{34}O_2$	270	88
14	15.286	3.15	cis-9-Hexadecenoic acid	$C_{16}H_{30}O_2$	254	96
15	15.413	21.76	n-Hexadecanoic acid (Palmitic acid)	$C_{16}H_{32}O_2$	256	96
16	15.55	0.97	Dodecanoyl Chloride (Lauric acid chloride)	$C_{12}H_{23}ClO$	218	75
17	15.761	0.21	Cholest-5-en-3-ol (3.beta.)-, carbonochloridate (Cholesterol, chloroformate)	$C_{28}H_{45}ClO_2$	448	76
18	16.021	1.08	Dodecanoic acid, 2-hydroxy-1-(hydroxymethyl)ethyl ester (Lauric acid .beta.-monogly)	$C_{15}H_{30}O_4$	274	82
19	16.137	1.14	Oxiraneundecanoic acid, 3-pentyl-, methyl ester, cis-	$C_{19}H_{36}O_3$	312	85
20	16.35	26.28	Octadec-9-enoic acid (cis-oleic acid)	$C_{18}H_{34}O_2$	282	95
21	16.439	5.82	Octadecanoic acid (Stearic acid)	$C_{18}H_{36}O_2$	284	94
22	16.542	0.25	9,12-Hexadecadienoic acid, methyl ester	$C_{17}H_{30}O_2$	266	78
23	16.723	0.21	9,12-Octadecadienoic acid (Z,Z)- (Linoleic acid)	$C_{18}H_{32}O_2$	280	91
24	17.004	0.86	Undecanal, 2-methyl-	$C_{12}H_{24}O$	184	81
25	17.345	0.31	Eicosanoic acid (Arachidic acid)	$C_{20}H_{40}O_2$	312	90
26	17.82	0.23	Octanoic acid, 2-dimethylaminoethyl ester	$C_{12}H_{25}NO_2$	215	82
27	17.901	0.13	cis-9-Hexadecenal	$C_{16}H_{30}O$	238	83
28	18.101	0.49	Hexadecanoic acid, 2-hydroxy-1-(hydroxymethyl)ethyl ester	$C_{19}H_{38}O_4$	330	95
29	18.55	0.46	Octadecanamide	$C_{18}H_{37}NO$	283	92
30	19.01	1.46	Oleoyl chloride (Oleic acid chloride)	$C_{18}H_{33}ClO$	300	92
31	19.163	0.61	9-Octadecenoic acid, 1,2,3-propanetriyl ester, (E,E,E)-	$C_{57}H_{104}O_6$	884	89
32	20.3	0.44	Octadecanoic acid, 2,3-bis[(1-Oxotetradecyl)Oxy]propyl ester	$C_{49}H_{94}O_6$	778	77
33	21.136	0.36	Cholesta-3,5-Diene	$C_{27}H_{44}$	368	90

Table S1. AWME3 chemical compositions detected by GC-MS.

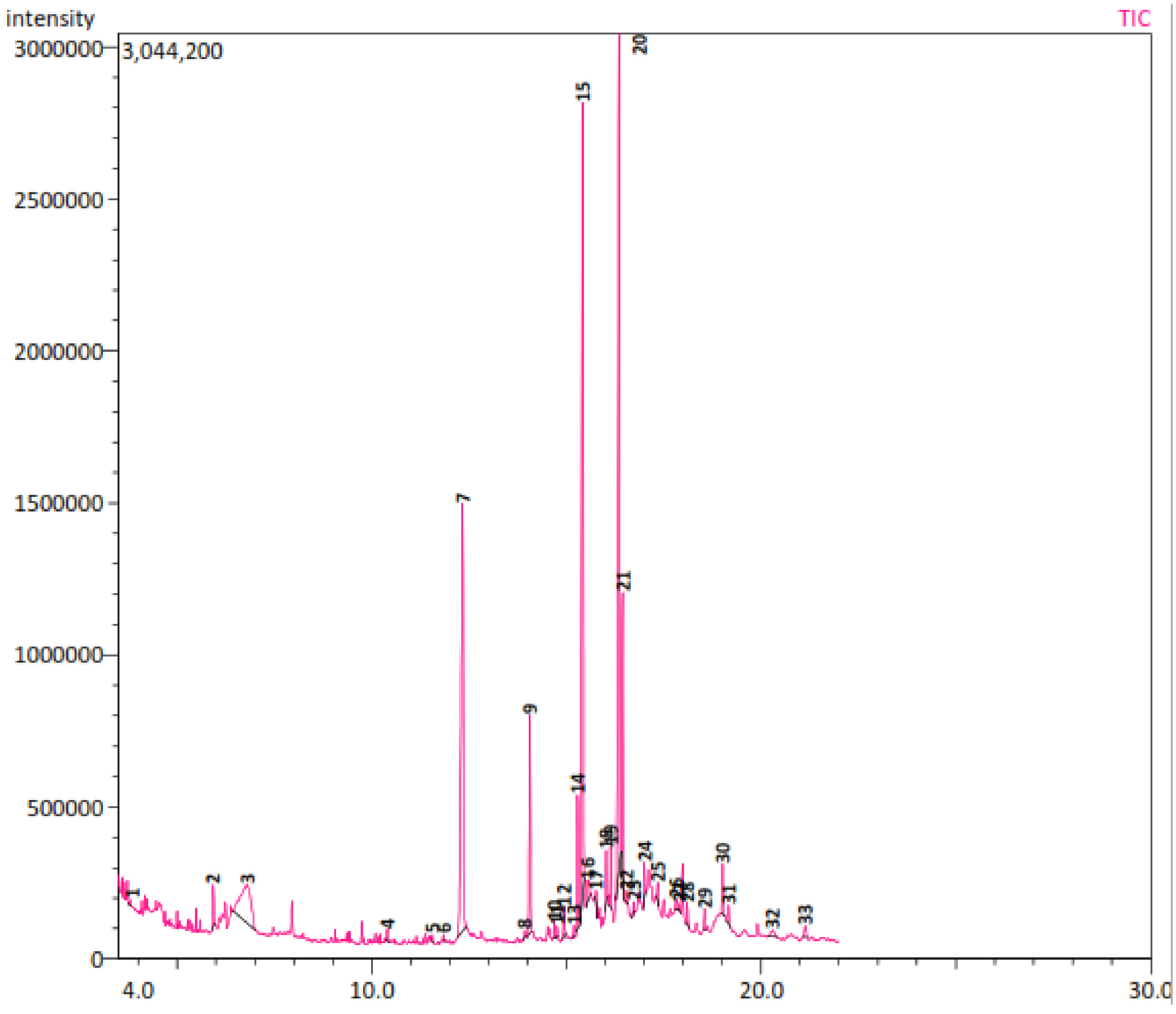

Fig. S1. GC-MS chromatogram of AWME3. The fatty acids profile of 33 compounds was identified by NIST-08 library.
